# MOF-Derived ZnS Nanodots/Ti_3_C_2_T_*x*_ MXene Hybrids Boosting Superior Lithium Storage Performance

**DOI:** 10.1007/s40820-021-00728-x

**Published:** 2021-09-26

**Authors:** Bin Cao, Huan Liu, Xin Zhang, Peng Zhang, Qizhen Zhu, Huiling Du, Lianli Wang, Rupeng Zhang, Bin Xu

**Affiliations:** 1 State Key Laboratory of Organic–Inorganic Composites, Beijing Key Laboratory of Electrochemical Process and Technology for Materials, Beijing University of Chemical Technology, Beijing, 100029 People’s Republic of China; 2 College of Materials Science and Engineering, Xi’an University of Science and Technology, Xi’an, 710054 People’s Republic of China

**Keywords:** Ti_3_C_2_T_*x*_ MXene, MOF, Interfacial interaction, Heterointerface, ZnS, Lithium-ion batteries

## Abstract

ZnS has great potentials as an anode for lithium storage because of its high theoretical capacity and resource abundance; however, the large volume expansion accompanied with structural collapse and low conductivity of ZnS cause severe capacity fading and inferior rate capability during lithium storage. Herein, 0D-2D ZnS nanodots/Ti_3_C_2_T_*x*_ MXene hybrids are prepared by anchoring ZnS nanodots on Ti_3_C_2_T_*x*_ MXene nanosheets through coordination modulation between MXene and MOF precursor (ZIF-8) followed with sulfidation. The MXene substrate coupled with the ZnS nanodots can synergistically accommodate volume variation of ZnS over charge–discharge to realize stable cyclability. As revealed by XPS characterizations and DFT calculations, the strong interfacial interaction between ZnS nanodots and MXene nanosheets can boost fast electron/lithium-ion transfer to achieve excellent electrochemical activity and kinetics for lithium storage. Thereby, the as-prepared ZnS nanodots/MXene hybrid exhibits a high capacity of 726.8 mAh g^−1^ at 30 mA g^−1^, superior cyclic stability (462.8 mAh g^−1^ after 1000 cycles at 0.5 A g^−1^), and excellent rate performance. The present results provide new insights into the understanding of the lithium storage mechanism of ZnS and the revealing of the effects of interfacial interaction on lithium storage performance enhancement.

**Supplementary Information:**

The online version contains supplementary material available at 10.1007/s40820-021-00728-x.

## Highlights

- The unique 0D-2D ZnS nanodots/Ti_3_C_2_T_*x*_ MXene hybrids with strong interfacial interaction enable to achieve stable cyclability and excellent rate performance for lithium storage.
- The lithium storage mechanism of ZnS is clarified and new insights into phase transition mechanism are proposed.
- The strong interfacial interaction between ZnS nanodots and MXene nanosheets at the ZnS-MXene heterointerface exhibits high lithium adsorption capability, enhanced interfacial electron transfer, and low lithium diffusion energy barrier.

**Supplementary Information:**

The online version contains supplementary material available at 10.1007/s40820-021-00728-x.

## Graphical abstract

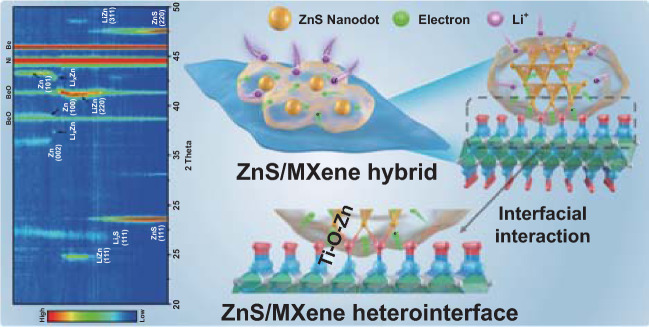

## Introduction

Electrochemical energy storage is a rapidly growing research field due to the ever-increasing requirements for smart grids and electric/hybrid vehicles. Lithium-ion batteries (LIBs) are currently the dominant energy storage system due to their high energy and power density, long cycle life, and low self-discharge rate [1, 2]. Nevertheless, the present LIBs technology faces challenges to achieve a higher energy density and power output because the currently used graphite anodes have a low theoretical capacity (372 mAh g^−1^) and unsatisfied rate performance. In addition, the low lithiation potential (~ 0.1 V *vs*. Li^+^/Li) of graphite enables the risk of lithium dendrites formation, which may trigger internal short and thus lead to safety concern [3, 4]. Transition metal sulfides (TMSs) [5–7], such as MoS_2_ [8, 9], SnS_2_ [10, 11], CoS [12], and ZnS [13], have received considerable attention as promising candidate anode materials to replace graphite on account of their high specific capacity and moderate working potential. Among TMSs, ZnS is one of the most attractive anode candidates for lithium storage because of its high theoretical lithium storage capacity, non-toxicity, and low cost. Based on the conversion and alloying reactions from ZnS to LiZn alloy and Li_2_S, a high theoretical specific capacity of 825 mAh g^−1^ can be achieved [14, 15]. Nevertheless, bulk ZnS suffers drastic capacity fading, which is caused by its substantial volume change during lithium storage and the consequent pulverization of ZnS particles. In addition, the low conductivity of ZnS leads to sluggish kinetics for lithium/electron transfer, which limits the rate performance for lithium storage. It is worth noting that the ZnS materials lacking conductive matrix and electron transfer pathway also usually suffers from unsatisfactory electrochemical activities [16, 17].

Recently, significant efforts have been devoted to improve the electrochemical performance of ZnS by using nanosized ZnS or designing nanostructures, such as ZnS quantum dots [13], ZnS nanotubes [18], and ZnS nanocrystals [19], which is an effective way to reduce the absolute volume expansion and shorten the diffusion pathway in solid phase during lithiation/delithiation. Anchoring ZnS on conductive matrix is another effective way to improve the electrochemical performance of ZnS as it can not only improve the conductivity but also accommodate the volume change. Several conductive matrixes including graphene [5, 6, 16], porous carbon [17, 20, 21], and carbon nanotubes [22] were adopted to combine with ZnS for improving electrochemical performance. MXenes, a new family of the 2D materials, have already been proved to be promising candidate as electrode materials for electrochemical energy storage devices [23–31], such as LIBs and supercapacitors. Among the large family of MXenes, Ti_3_C_2_T_*x*_ [32] is the most widely studied one for electrochemical energy storage owing to its high electronic conductivity (up to 2.4 × 10^4^ S cm^−1^) [33], hydrophilic surfaces and high density [34]. The theoretical conductivity of the Ti_3_C_2_T_*x*_ MXene is even higher than carbon nanotubes and graphene because of its metal carbide core layers [35]. Hence, using MXene as an excellent conductive matrix is very effective to improve the electrochemical performance of active materials, such as TMS and other active materials (such as SnO_2_ [36, 37], Co_3_O_4_ [38], P [27, 39, 40], and Si [41, 42]) with low intrinsic conductivity. Inspired by the above discussion, Ti_3_C_2_T_*x*_ MXene is considered as a superior conducting substrate to improve the electrochemical performances of ZnS.

In this work, the 0D-2D ZnS nanodots/Ti_3_C_2_T_*x*_ MXene nanosheets hybrids (ZnSMX) with excellent lithium storage performance were prepared by coordination modulation between MXene and ZIF-8 precursor followed with sulfidation. In ZnSMX hybrids, the nanodot morphology of ZnS is beneficial to reduce the lithium diffusion distance in solid phase and enhance the electrochemical kinetics. Furthermore, the Ti_3_C_2_T_*x*_ MXene nanosheets serving as 2D substrate not only provide fast electron transfer pathways, but also prevent the aggregation of ZnS nanodots and accommodate the volume change of ZnS during charge–discharge. More importantly, ZnS nanodots can be in situ locked tightly on the surface of 2D MXene nanosheets through interfacial Ti–O–Zn bond, thereby effectively suppressing the detachment of ZnS nanodots from the conducting MXene substrate. According to XPS characterizations and DFT calculation results, the charge density trends to accumulate at ZnS side in ZnS-MXene heterointerface, which indicates strong interfacial interaction and may enhance the electron mobility and electronic conductivity of ZnS. Moreover, ZnS-MXene heterointerface exhibits strong lithium adsorption capability and low diffusion energy barrier, which favors for achieving excellent electrochemical performances. Benefiting from these advantages, the 0D-2D ZnS/MXene hybrid shows a high reversible capacity of 726.8 mAh g^−1^ at 30 mA g^−1^, and long-term cycling stability of 462.8 mAh g^−1^ after 1000 cycles at 0.5 A g^−1^ without obvious capacity fading. The results imply that optimizing the interfacial interaction between ZnS nanodots and Ti_3_C_2_T_*x*_ nanosheets plays an important role for boosting lithium storage performances.

## Experimental Section

### Synthesis of Ti_3_C_2_T_***x***_ MXene Nanosheets

1.0 g of Ti_3_AlC_2_ (11Technology Co., Ltd) was slowly added to a mixed solution of 0.99 g LiF (Alfa Aesar, 98.5%) and 10 mL HCl (12 M), and the mixture was stirred at 35 °C for 24 h. Afterward, the multilayered Ti_3_C_2_T_*x*_ was carefully washed and centrifuged with deionized water for 5 times at a speed of 5000 rpm until the pH value of the supernatant reached ~ 6. The resulting Ti_3_C_2_T_*x*_ suspension was mixed with 200 mL deionized water and ultrasonicated for 1 h under argon atmosphere. Followed by centrifugation at 3500 rpm for 1 h, the aqueous suspension containing MXene nanosheets was obtained. The water in aqueous MXene dispersion was replaced with methanol by solvent exchange method. Finally, Ti_3_C_2_T_*x*_ MXene methanol dispersion was obtained for later use.

### Synthesis of ZIF-8/MXene

Zinc nitrate hexahydrate methanol solution (0.9 mmol Zn(NO_3_)_2_·6H_2_O, in 20 mL methanol) was added into the Ti_3_C_2_T_*x*_ methanol dispersion (25 mL, 2 mg mL^−1^) under stirring for 30 min. Then, 2-methylimidazole (2-MeIM) was dissolved in 20 mL methanol. Subsequently, the 2-MeIM methanol solution was poured into the as-prepared zinc nitrate hexahydrate/Ti_3_C_2_T_*x*_ MXene methanol dispersion (Zn:2-MeIM = 1:16 M) under magnetic stirring for 1 h at room temperature. After filtrating the suspension on Celgard 3501 membrane and further washing with methanol three times, the sample labeled as ZIF-8/MXene-0.9 was obtained and then dried at 80 °C in a vacuum oven overnight. The ZIF-8/MXene-2 precursor with a higher ZIF-8 content was prepared by using 2 mmol Zn(NO_3_)_2_·6H_2_O under similar preparation conditions.

### Synthesis of ZnS/MXene Hybrids

The ZIF-8/MXene-(0.9, 2) precursor and thioacetamide (TAA, 10 mmol) were added to a methanol/water mixed solution (volume ratio of 100 mL/10 mL), and stirred under an argon atmosphere for 30 min. Subsequently, the mixed solution was refluxed and stirred at 60 °C for 6 h. The sulfur substitution reaction was protected under nitrogen atmosphere until the reaction was finished. Then, the black solid resultant was collected by filtration, washed with deionized water and dried. The mass ratio of ZnS derived from the ZIF-8/MXene-0.9 and ZIF-8/MXene-2 was calculated as 64 and 80 wt%, respectively. Thus, the corresponding ZnS/MXene hybrids were denoted as ZnSMX64 and ZnSMX80. For comparison, the pure ZnS was prepared with the same method just without adding MXene dispersion.

### Material Characterization

The morphology and structure of the samples were characterized using scanning electron microscope (SEM, Hitachi S4800), transmission electron microscopy (TEM, Hitachi HT7700) and atomic force microscopy (AFM, Bruker edge). The XRD patterns of the samples were collected by using Bruker D8 Advanced XRD with Cu Kα radiation source. Raman spectra were obtained using a Raman spectrometer (Renishaw inVia Reflex with 633 nm laser excitation). XPS measurements were taken on a Thermo Fisher ESCALAB Xi^+^. The Zeta potential was conducted using a Zetasizer Nano ZSE apparatus. The specific surface area and porosity parameters were determined by the nitrogen sorption/desorption using a Micromeritics ASAP2460 analyzer.

### Electrochemical Measurements

The electrodes were prepared by mixing active material (80 wt%), conductive carbon (Super P, 10 wt%), and carboxymethylcellulose (CMC, 10 wt%) with water into an electrode slurry and then the slurry was cast onto a Cu foil and dried in a vacuum oven at 120 °C for 8 h. The mass loading of electrodes was controlled as 1 mg cm^−2^. Lithium foil and glass fiber membrane were used as the counter electrode and separator, respectively, and 1 M LiPF_6_ in ethylene carbonate (EC)/diethylcarbonate (DEC) (1:1 in volume) was used as the electrolyte. 2025 coin-type cells were assembled in an argon-filled glove box (with O_2_ and H_2_O < 0.1 ppm) to evaluate the electrochemical performance on Neware batteries testing systems (CT-4008T-5V50mA-164, Shenzhen, China). The capacity was calculated based on the mass of the ZnS/MXene hybrids. Cyclic voltammetry (CV) curves were collected at different scan rates between 0.01 and 2.5 V on a DH7000 electrochemistry workstation (Jiangsu Donghua Testing Technology Co., Ltd.). The electrochemical impedance spectroscopy (EIS) measurements were taken within a frequency range of 100 kHz–0.01 Hz with an amplitude of 10 mV. The GITT measurements were taken at a current density of 100 mA g^−1^ for 15 min followed by relaxation of 2 h. The in situ XRD measurement was taken on Bruker D8 (Cu Kα radiation, 40 kV and 40 mA) with an in situ cell equipped with Be window to allow X-ray passage (designed by Tianjin AIDA hengsheng Science and Technology Development Co., Ltd.) under a scan rate of 4° min^−1^. The scan range was 2*θ* = 20–50°, and the corresponding galvanostatic discharge–charge cycling was conducted at a current density of 100 mA g^−1^.

## Results and Discussion

**Fig. 1:**
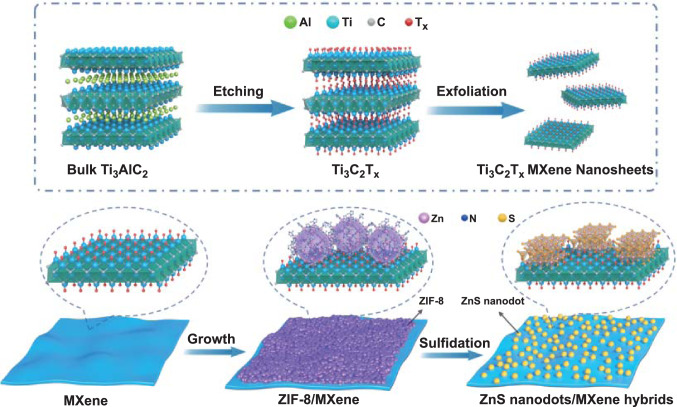
Schematic illustration for the synthesis of ZnS/MXene hybrids

The preparation of ZnS/MXene hybrids is schematically illustrated in Fig. 1. Initially, Ti_3_AlC_2_ powder was etched by LiF and HCl followed with exfoliation to achieve Ti_3_C_2_T_*x*_ MXene nanosheets with a lateral size of 500 nm (Fig. S1). The zeta potential of the prepared MXene sheets is − 36 mV (Fig. S2), which is ascribed to the functional groups (e.g., –F, –O, –OH) on the surface. The Ti_3_C_2_T_*x*_ MXene was used as substrate for in situ nucleation and growth of the ZIF-8. After adding zinc nitrate hexahydrate methanol solution into MXene methanol dispersion, the positively charged Zn^2+^ was electrostatically absorbed on the surface of MXene nanosheets, and then, the Zn^2+^ acted as the central site to coordinate with the organic linker 2-MeIM, resulting in the formation of ZIF-8/MXene hybrids. The subsequent sulfidation process was completed in methanol/water mixture with TAA under refluxing at 60 °C under a nitrogen atmosphere. The weak coordination bond between 2-MeIM and Zn^2+^ ion was easily broken [13], and thus, the exposed Zn^2+^ can recombine with the S^2−^ produced by the TAA hydrolysis to form ZnS.

**Fig. 2:**
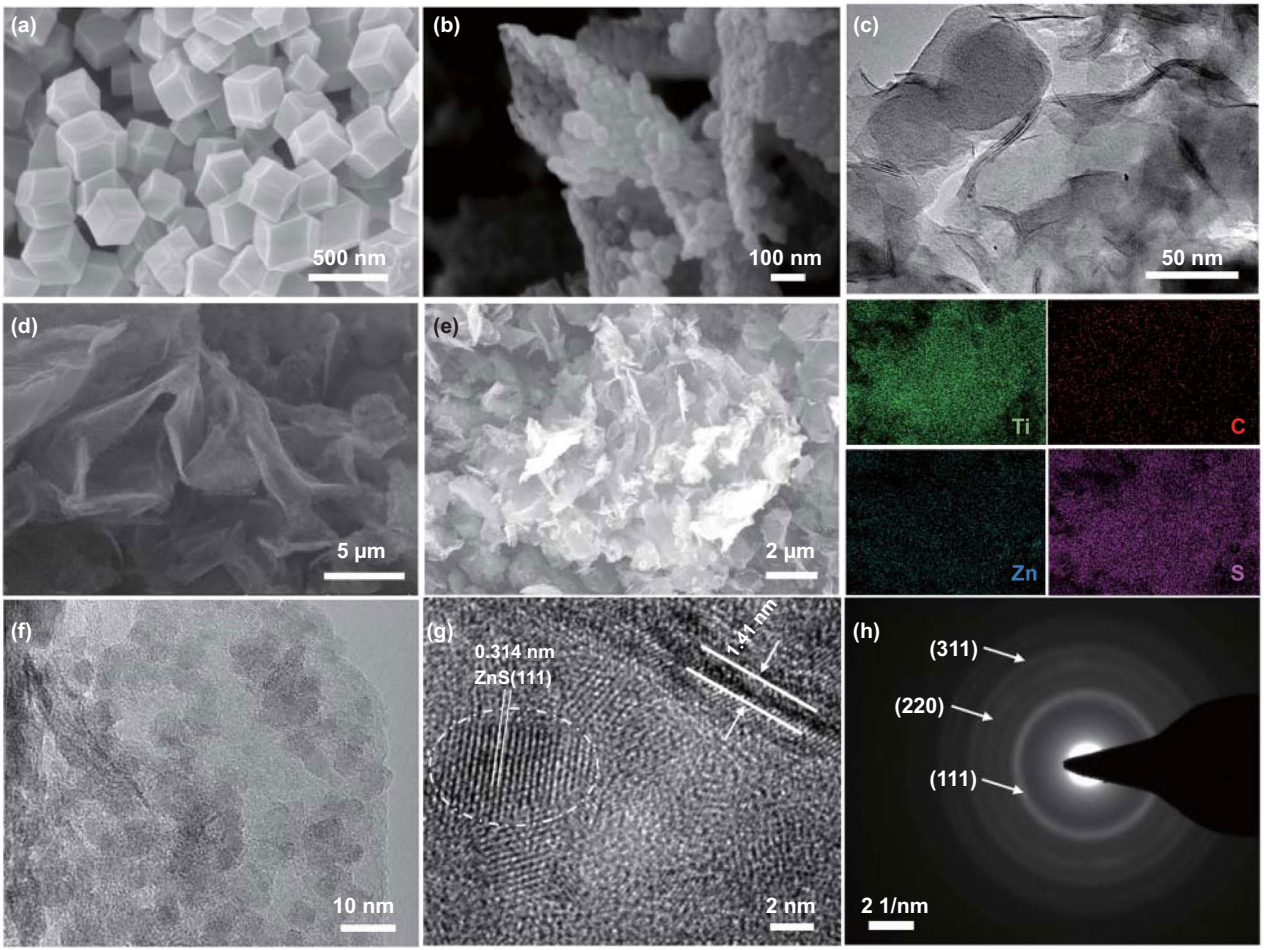
Morphology characterizations. SEM images of **a** ZIF-8 and **b** ZIF-8/MXene-0.9, and **c** TEM image of ZIF-8/MXene-0.9. **d** SEM image of ZnS/MX64 and **e** EDS elemental mapping of Ti, S, Zn and C of the corresponding SEM image of the ZnS/MX64. **f, g** HRTEM images of ZnS/MX64 and **h** the corresponding SAED pattern

The morphologies of ZIF-8, intermediate ZIF-8/MXene and the final product ZnS nanodots/MXene hybrids were characterized using SEM and TEM. SEM image (Fig. 2a) shows that the obtained ZIF-8 particles exhibit a typical rhombic dodecahedral shape with a homogeneous size of ~ 300 nm. In comparison with pure ZIF-8, ZIF-8 particles stabilized on the surface of MXene nanosheets exhibit smaller particle size of ~ 50 nm (Figs. 2b, c and S3a) in ZIF-8/MXene-0.9. With the decrease in the MXene content, the ZIF-8 particles in ZIF-8/MXene-2 accumulate excessively on the surface of the MXene nanosheets (Fig. S3b, c). In addition, the particle size of ZIF-8 in ZIF-8/MXene-2 is ~ 100 nm, which is bigger and closer to rhombic dodecahedral shape than that in ZIF-8/MXene-0.9. The above results indicate that the MXene nanosheets can act as the nucleation sites for the growth of ZIF-8.

Figure 2d shows that ZnSMX64 has open interconnected structure and the surface of MXene nanosheets is rough. It can be seen that the small particles are uniformly distributed on the surface of MXene nanosheets. Energy-dispersive spectroscopy (EDS) elemental mapping (Fig. 2e) of ZnSMX64 shows the uniform distribution of Zn, S, Ti, and C elements throughout the samples, suggesting the rough surface of the MXene nanosheet is composed of a large number of ZnS nanodots. HRTEM images (Fig. 2f, g) indicate that ZnS nanodots with particle sizes of 3–8 nm are uniformly and tightly anchored on the surface of 2D MXene nanosheets to form the 0D-2D structure. The lattice fringes with a d-spacing of 0.314 and 1.41 nm correspond to the (111) and (002) planes of ZnS [13] and MXene nanosheets [43] in the ZnSMX64, respectively. In addition, the hybridized boundary between ZnS nanodots and MXene nanosheets is a typical heterointerface structure, which may provide highly active sites for lithium adsorption and electrons transfer [6]. The corresponding selected area electron diffraction (SAED) pattern of ZnSMX64 in Fig. 2h displays dispersive diffraction rings related to (111), (220), (311) planes of ZnS. For ZnSMX80 (Fig. S4a–c), the ZnS nanoparticles agglomerate severely on the surface of MXene due to the higher mass loading of ZnS.

**Fig. 3:**
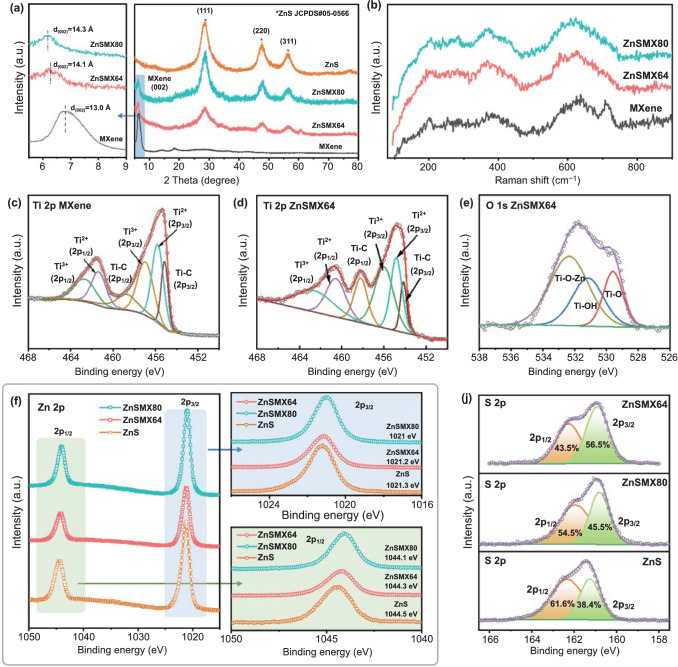
Structural characterizations of the ZnS nanodots/MXene hybrids.** a** XRD patterns, **b** Raman spectra, and **c, d** high-resolution Ti 2*p*, **e** O 1*s*, **f** Zn 2*p*, **j** S 2*p* XPS spectra

XRD was used to characterize the crystallinity and phase component of the samples. The XRD patterns of ZIF-8 (Fig. S5a) and MXene (Fig. 3a) are well agreed with previous reports [13, 30]. In diffraction pattern of MXene, the strong peak at 6.8° corresponds to (002) peak of Ti_3_C_2_T_*x*_ MXene film [44]. The XRD patterns of the ZIF-8/MXene-0.9 and ZIF-8/MXene-2 show characteristic peaks of ZIF-8 and MXene (Fig. S5b, c). However, after sulfidation, the XRD patterns (Fig. 3a) of ZnSMX64 and ZnSMX80 exhibit the characteristic peaks of ZnS (PDF No. 05-0566) and MXene, in which three diffraction peaks at 2*θ* of 28.6°, 47.5°, and 56.3° are assigned to (111), (220), and (311) planes of cubic structure ZnS [13, 45] and the peak round 6.2° is the characteristic (002) peak of MXene. This result indicates the coexistence of ZnS and MXene nanosheets after the sulfidation. Besides, the d_002_ of MXene in the ZnS/MXene hybrids increase to 14.1 Å for ZnSMX64 and 14.3 Å for ZnSMX80, larger than that of pure MXene (13 Å), which may be ascribed to the existence of methanol molecules in MXene nanosheets.

Raman spectra of the samples are presented in Figs. 3b and S6. The Raman spectrum of MXene shows peaks around 200, 387, 622, and 708 cm^−1^, corresponding to the typical characteristic vibrational modes for Ti_3_C_2_T_*x*_ MXene [46, 47]. As shown in Fig. S6, Raman spectra of ZIF-8/MXene composites mainly exhibit the characteristics of ZIF-8, which is ascribed to ZIF-8 anchoring on the surface of MXene nanosheets. For the ZnS/MXene hybrids, the peak at ~ 285 cm^−1^ is assigned to ZnS [48, 49]. Thus, the main characteristic peaks of ZnS/MXene hybrids (Fig. 3b) can be ascribed to MXene and ZnS species. Moreover, the characteristic peak (708 cm^−1^) representing the vibration of the C atoms in Ti_3_C_2_O(OH) [46] vanishes in the ZnS/MXene hybrids, implying that the surface of Ti_3_C_2_T_*x*_ MXene was hybridized with ZnS nanodots by dehydrogenation of hydroxyl functional group.

According to the XPS survey spectra, Zn and S peaks related to ZnS nanodots are clearly detected in the ZnS/MXene hybrids in contrast with pure Ti_3_C_2_T_*x*_ MXene (Fig. S7). The high-resolution C 1*s* spectrum of pure MXene (Fig. S8a) is fitted by three peaks at 281.8 eV (C–Ti), 285 eV (C–C/C = C), and 286.2 eV (C–O) [50], while for that of ZnS/MXene hybrids (Fig. S8b, c), it is evident that the C–Ti bond (281.8 eV) is originated from Ti_3_C_2_T_*x*_ MXene. The high-resolution Ti 2*p* XPS spectra of pristine Ti_3_C_2_T_*x*_ MXene (Fig. 3c) show multiple peaks as Ti–C (2*p*_*3/2*_ 455.2 eV and 2*p*_*1/2*_ 458.8 eV), Ti^2+^ (2*p*_*3/2*_ 455.8 eV and 2*p*_*1/2*_ 461.4 eV), and Ti^3+^ (2*p*_*3/2*_ 457.0 eV and 2*p*_*1/2*_ 462.8 4 V), which are consistent with the typical values reported for Ti_3_C_2_T_*x*_ MXene [1, 51]. The high-resolution Ti 2*p* XPS spectra of ZnS/MXene hybrids (Figs. 3d and S9) are similar with that of MXene, confirming the stability of MXene in ZnS/MXene hybrids without oxidation. Moreover, the interfaces interaction state of MXene and ZnS can be further investigated in O 1*s* XPS spectra. As shown in Fig. S10a, the O 1*s* region of MXene can be fitted into two peaks located at 530 eV (Ti–O) and 531.5 eV (Ti–OH) [39]. It is noteworthy that there is a new fitted peak at 532.3 eV in the ZnS/MXene hybrids, which may be attributed to Ti–O–Zn bonds [52] (Figs. 3e and S10b), indicating the formation of chemical bonds at the interface between ZnS and MXene. The formation of the Ti–O–Zn interfacial bonding is helpful to anchor ZnS nanodots on the MXene matrix tightly as well as strengthen the interaction between the two components.

The high-resolution Zn 2*p* spectra of the ZnS/MXene hybrids and ZnS are divided into one couple of peaks (Fig. 3f): the 2*p*_*1/2*_ and 2*p*_*3/2*_, which confirms the existence of Zn in the composites with the formation of Zn^2+^ [53]. Significantly, compared to pure ZnS, both the Zn 2*p*_*1/2*_ and Zn 2*p*_*3/2*_ peaks of the ZnS/MXene hybrids shift to lower binding energy. It suggests that a more electron-rich state of Zn 2*p* in the ZnS/MXene hybrids than that of pure ZnS, which may be attributed to the electron transfer from MXene to ZnS nanodots. In addition, as shown in Fig. 3j, the electron redistribution between ZnS nanodots and MXene nanosheets is further confirmed by the S 2*p* spectra with the characteristic peaks of 2*p*_*3/2*_ and 2*p*_*1/2*_ peaks in the ZnS phase [54]. Clearly, compared with that of pure ZnS and ZnSMX80, the S 2*p*_*3/2*_ peak of ZnSMX64 shifts to a lower binding energy with increased content, further proving the electron accumulation at ZnS nanodots [55]. Therefore, the above results demonstrate that the strong interfacial interactions with Ti–O–Zn bonds formed at the interfaces between ZnS nanodots and MXene nanosheets promotes the interface electron transfer, which are expected to significantly improve the electrochemical performance of the ZnS nanodots/MXene hybrids.

The specific surface areas and pore structures of ZnS, MXene and ZnS/MXene hybrids were studied by their N_2_ (77 K) adsorption/desorption isotherms. The isotherms of ZnS and ZnS/MXene hybrids exhibited in Fig. S11a are similar to typical IV hysteresis loops as defined by IUPAC [21, 56]. It can be seen that the specific surface areas of the ZnS/MXene hybrids (161.3 m^2^ g^−1^ for ZnSMX64, 153.4 m^2^ g^−1^ for ZnSMX80) are higher than those of the ZnS (141.2 m^2^ g^−1^) and MXene (19.0 m^2^ g^−1^). Moreover, the pore volume of ZnSMX64 (0.163 cm^3^ g^−1^) and ZnSMX80 (0.145 cm^3^ g^−1^) are much higher than those of ZnS (0.109 cm^3^ g^−1^) and MXene (0.078 cm^3^ g^−1^). The larger surface area and pore volume of ZnS/MXene hybrids are expected to synergistically promote fast ion diffusion and accommodate substantial volume changes. Figure S11b shows the pore size distributions of the ZnS, MXene and ZnS/MXene hybrids. The abundant mesopores (2–10 nm) are observed in the ZnS/MXene hybrids, which is beneficial for facilitating the transport of Li^+^, thus improving their rate performance.

**Fig. 4:**
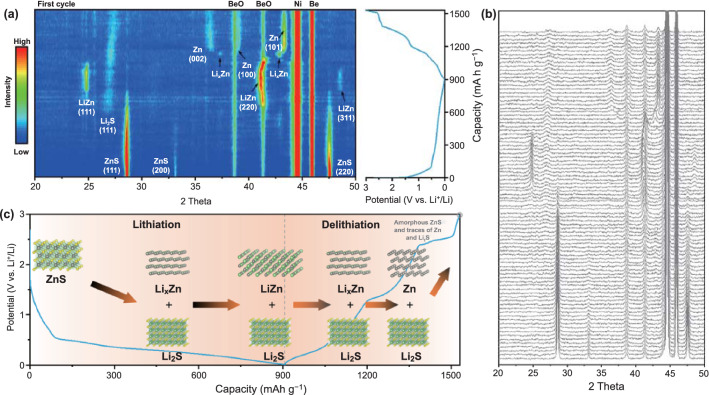
In situ XRD characterization of commercial ZnS anodes during lithium storage. **a** Contour plot of in situ XRD characterization with corresponding voltage profile and **b** diffraction patterns for CZnS anode in the first cycle. **c** Schematic illustration of the phase evolution of the CZnS in the first lithiation–delithiation cycle

In order to shed light on the phase evolution of ZnS during lithium storage, the in situ XRD characterization was carried out for commercial ZnS (CZnS) anodes. In comparison with the XRD patterns of the as-prepared ZnS/MXene samples, the XRD pattern (Fig. S12) of CZnS shows a similar crystal structure but larger crystal size. The electrochemical behavior of CZnS was also evaluated by CV (Fig. S13), which shows undesirable electrochemical reversibility similar to that of ZnS prepared in this work. The phase evolution of CZnS during the first lithium storage cycle was shown in the contour plot with corresponding voltage profiles (Fig. 4a) and diffraction patterns (Fig. 4b). The intensity of characteristic peaks of ZnS gradually decreases upon lithiation due to the conversion reaction of ZnS with lithium. Subsequently, the existence of Li_2_S is confirmed by the emergence of (111) peak of Li_2_S while Zn metal and Li_x_Zn are not observed. At full lithiation state, both LiZn and Li_2_S are observed as final products. During delithiation, the characteristic peaks of LiZn gradually disappear until the emergence of Li_x_Zn (such as LiZn_4_) [57]. Immediately, the phase of Li_x_Zn vanishes while the phase of Zn metal appeares. The intensity of (101) peak of Zn firstly exhibits increases due to the accumulation of Zn metal, as lithium is totally extracted from Li–Zn alloy. Subsequently, the intensity of (101) peak of Zn gradually decreases upon delithiation due to the consumption of Zn in the conversion reaction. At full delithiation state, ZnS phase cannot be observed, while the weak peaks of Zn and Li_2_S still remain, which is ascribed to the amorphous nature of the new generated ZnS and the poor reversibility of the conversion reaction, respectively [57]. Based on the above results, the phase evolution of ZnS during first lithiation–delithiation cycle is illustrated in (Fig. 4c). In the second cycle, the phase evolution is also shown in the contour plot (Fig. S14) and the corresponding diffraction patterns (Fig. S15). During lithiation, the amorphous ZnS is firstly converted to Zn and Li_2_S, leading to the increasing intensity of characteristic peak of Zn. Afterward, Li_x_Zn was formed during further lithiation due to the alloying reaction of Zn with lithium. At full lithiation state, the LiZn phase is finally formed, but its peak intensity is weaker than that in the first cycle, suggesting poor lithium storage reversibility. During delithiation, a reverse process is observed. According to the above results, the following lithium storage mechanism of ZnS is proposed.

- First cycle: ZnS → amorphous Li_x_Zn + Li_2_S → LiZn + Li_2_S (full lithiation state) → Li_x_Zn + Li_2_S → Zn + Li_2_S → amorphous ZnS + traces of Zn and Li_2_S.
- Second cycle: amorphous ZnS + traces of Zn and Li_2_S → Zn + Li_2_S → Li_x_Zn + Li_2_S → LiZn + Li_2_S (full lithiation state) → Li_x_Zn + Li_2_S → Zn + Li_2_S → amorphous ZnS + traces of Zn and Li_2_S.

Based on the above analysis, the lithium storage mechanism of CZnS can be simply described as following: the ZnS is converted into LiZn and Li_2_S during lithiation; however, the resultant LiZn and Li_2_S incompletely recombine in delithiation, causing the presence of traces of Zn and Li_2_S, which reveal the poor reversibility of ZnS during lithium storage. In the second cycle, the irreversible phase transition occurs in ZnS more severely. The poor reversibility is the main reason for the quick capacity decay during cycling for CZnS (Fig. S16), which should be ascribed to the large volume expansion of ZnS as well as the consequent electrode pulverization (Fig. S17). Therefore, in order to overcome this problem and improve its cycle life, ZnS nanodots were prepared and further in situ riveted on MXene substrate to improve the lithium storage reversibility.

**Fig. 5:**
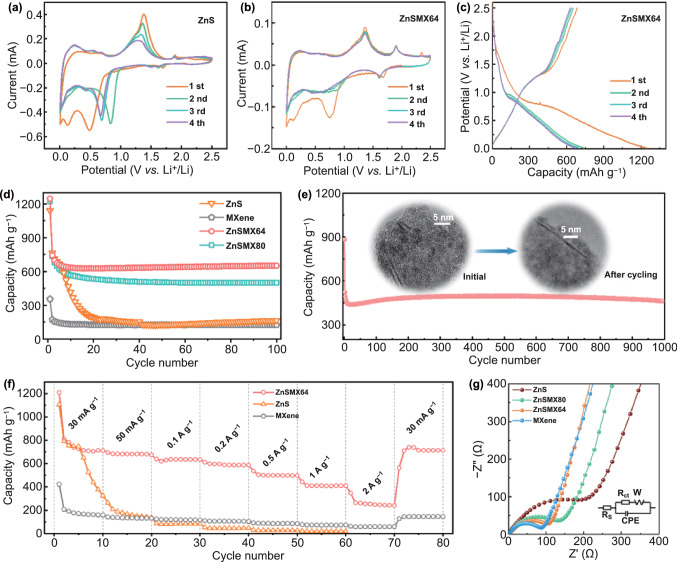
Lithium storage performance. CV curves of as-prepared **a** ZnS and **b** ZnSMX64 at a scan rate of 0.1 mV s^−1^. **c** Galvanostatic charge/discharge profiles of ZnSMX64 at 100 mA g^−1^. **d** Cyclability at 100 mA g^−1^, **e** long-term cycling performance of ZnSMX64 at 0.5 A g^−1^ with ex situ HRTEM images in the insets, **f** rate capabilities at different currents, and **g** Nyquist plots for ZnS and ZnS/MXene hybrids

The electrochemical behaviors of the ZnS and ZnS/MXene hybrids were evaluated by CV and galvanostatic charge–discharge test. The CV curves of pure ZnS, ZnSMX64, and ZnSMX80 at a scan rate of 0.1 mV s^−1^ are shown in Figs. 5a, b and S18. It can be seen that the pure ZnS and ZnS/MXene hybrids show similar electrochemical behaviors in the first cycle, but the redox peaks in the following cycles for the pure ZnS are quite different from those in the first cycle due to the low reversibility. From the CV curves of the ZnS/MXene hybrids (Figs. 5b and S18), we can perceive that the incorporation of MXene with ZnS nanodots can greatly improve the lithium storage reversibility. In particular, when introducing the appropriate amount of MXene, the CV curves of ZnSMX64 almost overlap after the first cycle, manifesting the highly stable structure and reversible reaction behavior during lithium storage cycling. Figures S19 and 5c show the first three charge–discharge curves of ZnS and ZnSMX64 electrodes at 100 mA g^−1^. Pure ZnS shows a lithiation/delithiation capacity of 1141.5/720.9 mAh g^−1^ with an initial Coulombic efficiency (ICE) of 63.2%. However, the gradual separated voltage profile from 1st to 4th cycle indicates unstable cyclability. The MXene delivers an initial lithiation/delithiation capacity of 355.3/166.3 mAh g^−1^, and no obvious voltage platform appears in the voltage range of 0.01–2.5 V (Fig. S20). For the ZnSMX64, the initial lithiation/delithiation capacity is 1246.9/683.2 mAh g^−1^ with the corresponding ICE of 54.8%. The higher capacity of ZnS/MXene hybrids than that of MXene is ascribed to the capacity contribution from ZnS nanodots. The comparison of the cycling performance of MXene, ZnS and ZnS/MXene hybrids at 100 mA g^−1^ is shown in Fig. 5d. For pure ZnS, the lithiation capacity decreases sharply to 190 mAh g^−1^ after 20 cycles. The rapid capacity fading may be ascribed to the pulverization of ZnS due to its large volume change during charge–discharge [58]. In contrast, the ZnSMX64 exhibits the best cycle stability and a high capacity of 650.6 mAh g^−1^ is remained after 100 cycles. Figure S21 shows the voltage profiles of ZnSMX64 under different cycles at a current density of 100 mA g^−1^. The voltage profiles almost overlap even after 100 cycles. This result indicates that the introducing of MXene is powerful to improve the cyclability of the ZnS/MXene hybrids electrodes, which agrees with the CV results mentioned above. It is noteworthy that ZnSMX64 exhibits better cyclability than ZnSMX80 anode, suggesting that the superfluous active ZnS will sacrifice its cycling performance. The insufficient MXene nanosheets in ZnSMX80 may not accommodate the substantial volume variation of agglomerated ZnS nanoparticles during lithium storage cycling. Moreover, the long-term cycling performance of ZnSMX64 at 0.5 A g^−1^ is shown in Fig. 5e. The ZnSMX64 exhibits a highly stable capacity of 462.8 mAh g^−1^ without obvious decay after 1000 cycles. From ex situ HRTEM characterization result, there is no structural collapse after 1000 cycles for ZnSMX64 electrode as shown in the insets of Fig. 5e. Therefore, such stable cyclability may be attributed to the rationally designed nanostructure of ZnS nanodots onto MXene substrate and the strong interfacial interaction between them.

Apart from the cycling performance, rate capability is also key factor to evaluate the potential value of the ZnS/MXene hybrids as LIBs anodes. Figure 5f shows the rate capability of all the samples at different current densities. The pure ZnS exhibits very poor rate performance with a capacity of 19.3 mAh g^−1^ at 1.0 A g^−1^. However, the ZnSMX64 anode exhibits excellent rate performance, especially under high current densities, which delivers reversible capacities of 726.8, 680.9, 635.1, 588.1, 497.7, 408.8, and 252.5 mAh g^−1^ at 0.03, 0.05, 0.1, 0.2, 0.5, 1, and 2 A g^−1^, respectively. Noticeably, a high capacity of 712.5 mAh g^−1^ can be retained when the current density is back to 0.03 A g^−1^. Moreover, EIS was used to investigate the charge transfer kinetics of the ZnS/MXene hybrids. As shown in Fig. 5g, the Nyquist plots consist of a semicircle in high and middle frequencies and a straight line at low frequency. The semicircle corresponds to the impedance of charge transfer, and the straight line is assigned to Li diffusion in electrodes [21]. Specifically, the Nyquist plot of MXene shows the smallest semicircle, indicating the lowest charge transfer resistance. In comparison with pure ZnS anode, both ZnSMX64 and ZnSMX80 show a smaller semicircle, indicating that the introducing MXene into ZnS nanodots can reduce charge transfer resistance and promote charge transfer kinetics. The semicircle of ZnSMX64 anodes is smaller than that of pure ZnS anode and ZnSMX80, suggesting the best charge transfer kinetics.

**Fig. 6:**
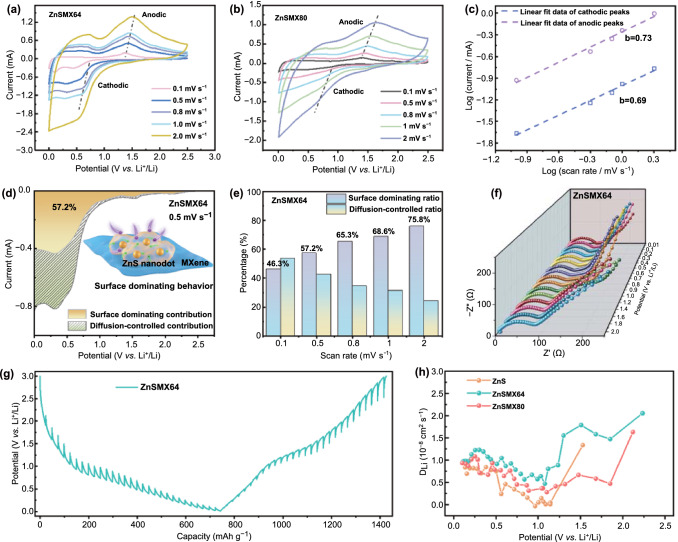
Electrochemical kinetics for the ZnS/MXene hybrids. CV curves of **a** ZnSMX64 and **b** ZnSMX80 at different sweep rates from 0.1 to 2.0 m V^−1^, and **c** the plots of log *i* versus log *υ*. **d** CV curve of ZnSMX64 with the surface dominating capacity contribution for cathodic process at 0.5 mV s^−1^, and **e** the proportion of capacity contributions at different scan rates. **f** In situ EIS characterization of ZnSMX64 at different lithiation states between 2.0 and 0.01 V. **g** GITT potential profile of the ZnSMX64 and **h** the Li^+^ diffusion coefficients in lithiation process

To explore the lithium storage behavior of the ZnS/MXene hybrids, CV curves were measured at different scan rates from 0.1 to 2 mV s^−1^ (Fig. 6a, b). Compared with ZnSMX80, ZnSMX64 has smaller redox potential differences (0.6 and 1.3 V) at 2 mV s^−1^, indicating weaker polarization and enhanced electrochemical kinetics. Furthermore, the electrochemical kinetics of ZnSMX64 is analyzed by power-law relationship [59] between measured current and sweep rate according to Eqs. (1) and (2):

1 $$i = a\upsilon^{b}$$

2 $$\log (i) = b\log (\upsilon ) + \log (a)$$

where *i* is current, *a* is a constant, *υ* is scan rate, and *b* is the slope of the log(*υ*)–log(*i*) plot. The *b* value close to 1 means a surface dominating behavior (non-diffusion-controlled process), while the *b* value of 0.5 is a diffusion-controlled process. As shown in Fig. 6c, the value of $b$ is 0.69 and 0.73 for the cathodic and anodic peak of ZnSMX64, respectively, revealing non-ignored surface dominating lithium storage behavior with good electrochemical kinetics. Furthermore, the measured current can be analyzed with Eq. (3):

3 $$i(\upsilon ) = k_{1} \upsilon + k_{2} \upsilon^{1/2}$$

where *k*_1_ and *k*_2_ are adjustable parameters, and *k*_1_*υ* and *k*_2_*υ*^*1/2*^ represent the surface dominating reaction current and diffusion-controlled reaction current, respectively [60]. As shown in Fig. 6d, the ZnSMX64 anode exhibits a surface dominating capacity contribution of 57.2% at a scan rate of 0.5 mV s^−1^, which is higher than that of ZnSMX80 (45.7%) (Fig. S22a). In addition, it can be observed that surface dominating contribution ratios increased with scan rates for both ZnSMX64 (Fig. 6e) and ZnSMX80 (Fig. S22b). The higher surface dominating capacity contributions in ZnSMX64 are benefit to exhibit a superior rate capability [20].

In situ EIS was performed to further elucidate the impedance properties of the ZnS/MXene hybrids with the ZnS anode as a comparison at different lithiation states (2.0–0.01 V). As shown in Figs. 6f and S22, the Nyquist plots exhibit gradually increased semicircle during the lithiation process, indicating increased charge transfer resistance. This result may be ascribed to the formation of SEI and gradually saturated lithiation state [6]. Moreover, the Nyquist plots of ZnSMX64 exhibit the smallest semicircle in the whole lithiation process compared with ZnSMX80 and ZnS anode, indicating the lowest charge transfer impedance and best charge transfer kinetics during lithiation. Furthermore, the galvanostatic intermittent titration technique (GITT) was used to investigate the Li^+^ diffusion properties as shown in Figs. 6g and S24 [61]. The apparent Li^+^ diffusion coefficients are calculated by Eq. S1. As shown in Fig. 6h, ZnSMX64 exhibits the higher apparent Li^+^ diffusion coefficients than ZnS and ZnSMX80, indicating the excellent electrochemical kinetics. In addition, the lithium storage performance of ZnS/MXene hybrids electrodes may be further improved if using MXene film instead of Cu foil as a current collector [62]. Therefore, the superior electrochemical performance of ZnSMX64 can be ascribed to its surface dominating lithium storage behavior with high reversibility and fast lithium diffusion capability.

**Fig. 7:**
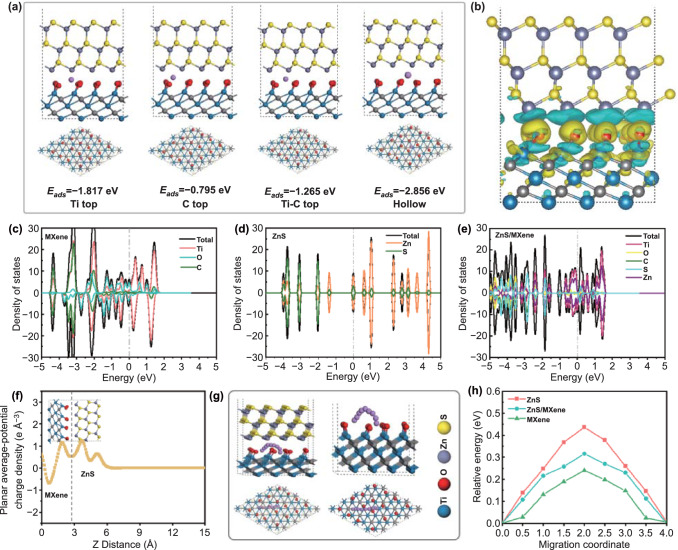
Theoretical simulation for lithium adsorption/migration at ZnS-Ti_3_C_2_T_*x*_ MXene heterointerface. **a** Lithium adsorption and the corresponding adsorption energy at Ti top, C top, Ti-C top and hollow site at ZnS (111)/Ti_3_C_2_T_*x*_ MXene heterointerface and **b** charge density differences of ZnS (111)/Ti_3_C_2_T_*x*_ MXene heterointerface. DOS plots (fermi levels are set as zero and indicated with dashed lines) of **c** Ti_3_C_2_T_*x*_, **d** ZnS, and **e** ZnS/Ti_3_C_2_T_*x*_ MXene. **f** Planar average potential charge density along the z axis (vertical direction) of ZnS (111)/Ti_3_C_2_T_*x*_ MXene heterointerface. **g** Lithium diffusion pathway and **h** the corresponding relative diffusion energy variation at ZnS (111)/Ti_3_C_2_T_*x*_ MXene heterointerface

Furthermore, the lithium adsorption and diffusion features at the ZnS/MXene heterointerface and the corresponding interfacial electronic structure were studied by density functional theory (DFT) calculations. First, three possible ZnS-Ti_3_C_2_T_*x*_ MXene heterointerface models with different ZnS orientation (Fig. S25a) were constructed, i.e., ZnS (111)-MXene, ZnS (101)-MXene, and ZnS (001)-MXene. Among them, the ZnS (111)-MXene heterointerface is the most stable one because of the strongest interfacial interaction (*E*_Binding_ = − 2.851 eV) (Fig. S25b). Therefore, the ZnS (111)-MXene heterointerface model was selected to evaluate lithium adsorption/migration features. The lithium adsorption energies at different sites in ZnS (111), MXene and ZnS (111)-MXene heterointerface were calculated, and the results are shown in Figs. S26, S27 and 7a, respectively. The ZnS (111)-MXene heterointerface exhibits the highest lithium adsorption capability due to the lowest average adsorption energies. Thus, the ZnS (111)-MXene heterointerface with strong lithium adsorption capability may function as active sites for improving lithium storage capacity. The interfacial interaction between ZnS and MXene was further clarified by the charge density differences, the density of states (DOS), and the planar average potential charge density. As shown in Fig. 7b, the charge density at the ZnS (111)-MXene heterointerface exhibits an accumulating tendency at the surface of ZnS, confirming a significant charge transfer phenomenon. According to the DOS plots of MXene to ZnS-MXene heterogeneous structure (Fig. 7c–e), the electron density near Fermi lever increases for ZnS (111)-MXene heterointerface compared with that of ZnS. Moreover, the planar average charge density along the direction of *z* axis of ZnS-MXene heterointerface is shown in Fig. 7f, which further proves the electron transfer from the electron-rich region (Ti_3_C_2_T_*x*_ MXene) to the electron-deficient region (ZnS). The interfacial electron migration from MXene to ZnS leads to interfacial charge redistribution and the corresponding formation of an interfacial electric field within the interfaces, which may effectively promote electron transfer and ion diffusion to achieve excellent electrochemical performance. Furthermore, lithium diffusion features were evaluated for MXene, ZnS (111) and ZnS (111)/MXene heterointerface. The lithium migration pathways from a hollow site to adjacent hollow sites are shown in Figs. 7g and S28. The diffusion relative energy is plotted in Fig. 7h. The lithium diffusion energy barrier of ZnS (111)/MXene heterointerface (0.32 eV) is lower than that of ZnS (0.44 eV), but higher than that of MXene (0.24 eV). This result indicates that MXene in ZnS (111)/MXene heterointerface can promote fast lithium migration. In comparison with ZnS, the lower diffusion energy barrier of ZnS (111)/MXene heterointerface enables fast lithium migration and excellent diffusion kinetics to boost rate performance. In brief, according to the DFT calculation results, MXene plays a critical role in promoting electron/mass transfer at ZnS (111)/MXene heterointerface to achieve excellent lithium storage performance.

**Fig. 8:**
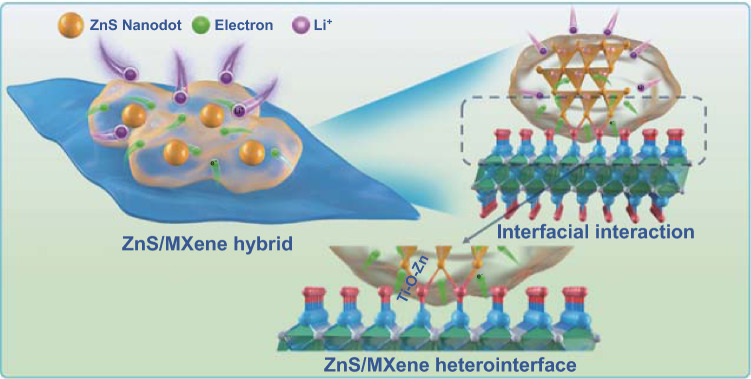
Schematic illustration for interfacial interaction at ZnS-MXene heterointerface boosting electron transfer and lithium diffusion

Based on the above results, we clearly demonstrate that the 0D-2D ZnS nanodots/MXene hybrid exhibits good lithium storage performance due to the surface dominating lithium storage behavior with excellent electrochemical kinetics. For ZnSMX64, the interfacial bonding of Ti–O–Zn between ZnS nanodots and MXene nanosheets can immobilize ZnS nanodots on the surface of MXene stably to prevent detachment of ZnS nanodots from conducting MXene matrix and boost the interfacial interaction to promote electron/mass transfer. At the ZnS-MXene heterointerface, the strong interfacial interaction will induce electron migration and charge density redistribution from MXene to ZnS. Correspondingly, the ZnS-MXene heterointerface exhibits a high lithium adsorption capability and low diffusion energy barrier. Therefore, the strong interfacial interaction between ZnS nanodots and MXene nanosheets makes ZnSMX64 to exhibit stable cyclability and superior rate performance. The possible interfacial interaction in ZnS-MXene heterointerface for boosting electron transfer and lithium adsorption/diffusion is schematically illustrated in Fig. 8. In comparison with pure ZnS, the improved lithium storage performance including stable cyclability and high rate performance of ZnSMX64 is ascribed to its unique 0D-2D structure, appropriate ZnS nanodots loading, and enhanced interfacial electron/mass transfer, which enable effective accommodation of substantial volume variations, stable electron conduction, and fast lithium diffusion.

## Conclusions

In summary, 0D-2D ZnS nanodots/Ti_3_C_2_T_*x*_ MXene hybrids were prepared by sulfidation the ZIF-8/MXene precursors to achieve excellent lithium storage performance. Different from the bulk ZnS with poor lithium storage performance due to substantial volume variation and low lithium storage reversibility, the ZnS nanodots/Ti_3_C_2_T_*x*_ MXene hybrids with the unique 0D-2D structure and strong interfacial interaction can enable immobilization of ZnS nanodots on MXene substrate, effective accommodation of substantial volume variations, stable electron conduction, and fast lithium diffusion. The as-prepared ZnSMX64 exhibits exceptional lithium storage performance with superior cycle stability (462.8 mAh g^−1^ after 1000 cycles at 0.5 A g^−1^ without obvious capacity fading) and rate capability (252.5 mAh g^−1^ at 2.0 A g^−1^), which is ascribed to the surface dominating lithium storage behavior with excellent electrochemical kinetics. DFT calculation result further elucidates the strong lithium adsorption capability, enhanced interfacial electron transfer, and low diffusion energy barrier at the ZnS-MXene heterointerface. Thus, designing a 0D-2D structure by anchoring the nanosized metal sulfide onto MXene nanosheets with strong interfacial interaction is a promising strategy to boost lithium storage performance, which can be extended to other metal dichalcogenides for energy storage application. Our work also deepens the understanding of electrochemical lithium storage mechanisms of ZnS-based anodes and the effect of interfacial interaction on the electron/mass transfer at heterointerface.

## Supplementary Information

Below is the link to the electronic supplementary material.

Supplementary file1 (PDF 1640 kb)
