# Supplementary material for: MOF-Derived ZnS Nanodots/Ti3C2Tx MXene Hybrids Boosting Superior Lithium Storage Performance
Source: Nanomicro Lett. 2021 Sep 26;13:202. doi: 10.1007/s40820-021-00728-x (PMC8473522; doi:10.1007/s40820-021-00728-x)
Supplement: Supplementary file 1 — Supplementary file1 (PDF 1640 kb) [file 40820_2021_728_MOESM1_ESM.pdf]

Supporting Information for

MOF-Derived ZnS Nanodots/$Ti_3C_2T_x$ MXene Hybrids Boosting Superior Lithium Storage Performance

Bin Cao[1], Huan Liu[1, 2, *], Xin Zhang[1], Peng Zhang[1], Qizhen Zhu[1], Huiling Du[2], Lianli Wang[2], Rupeng Zhang[1], and Bin Xu[1, *]

[1]State Key Laboratory of Organic–Inorganic Composites, Beijing Key Laboratory of Electrochemical Process and Technology for Materials, Beijing University of Chemical Technology, Beijing 100029, People's Republic of China

[2]College of Materials Science and Engineering, Xi'an University of Science and Technology, Xi'an 710054, People's Republic of China

*Corresponding authors. E-mail: huanliu@xust.edu.cn (H. Liu), xubin@mail.buct.edu.cn (B. Xu)

S1 DFT Calculation Methods

We have employed the Vienna ab initio package (VASP) [S1, S2] to perform all spin-polarization density functional theory (DFT) calculations within the generalized gradient approximation (GGA) using the Perdew-Burke-Ernzerhof (PBE) [S3] formulation. We have chosen the projected augmented wave (PAW) potentials [S4] to describe the ionic cores and take valence electrons into account using a plane wave basis set with a kinetic energy cutoff of 400 eV. Partial occupancies of the Kohn −Sham orbitals were allowed using the Gaussian smearing method and a width of 0.05 eV. The electronic energy was considered self-consistent when the energy change was smaller than 10^{-6} eV. A geometry optimization was considered convergent when the energy change was smaller than 0.05 eV Å^{-1}. In addition, for the Ti atoms, the U schemes need to be applied, and the U has been set as 3.2 eV. Finally, the adsorption energies (E_{ads}) were calculated as $E_{ads}= E_{ad/sub} - E_{ad} - E_{sub}$, where $E_{ad/sub}$, E_{ad}, and E_{sub} are the total energies of the optimized adsorbate/substrate system, the adsorbate, and the clean substrate, respectively. Moreover, Li ions migration barrier energies had been evaluated using the climbing nudged elastic band (CI-NEB) methods. In our calculation, the interface binding energy can be obtained by the equation: $E_{binding}= (E_{total} - E_1 - E_2)$, where the E_{total} is the energy of interface structure, E_1 and E_2 is the energy of the surfaces.

S2 Supplementary Figures

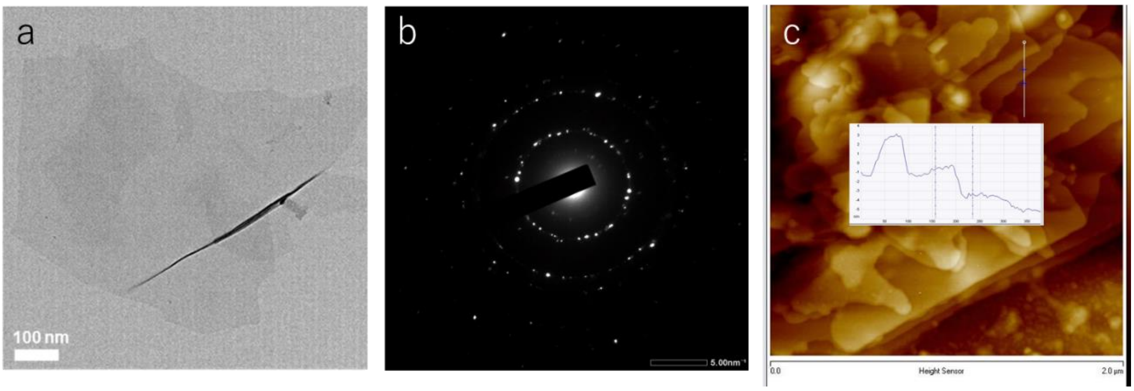

Fig. S1 TEM image (**a**) with the corresponding selected area electron diffraction pattern (**b**), and atomic force microscope image (**c**) of $Ti_3C_2T_x$ MXene nanosheets

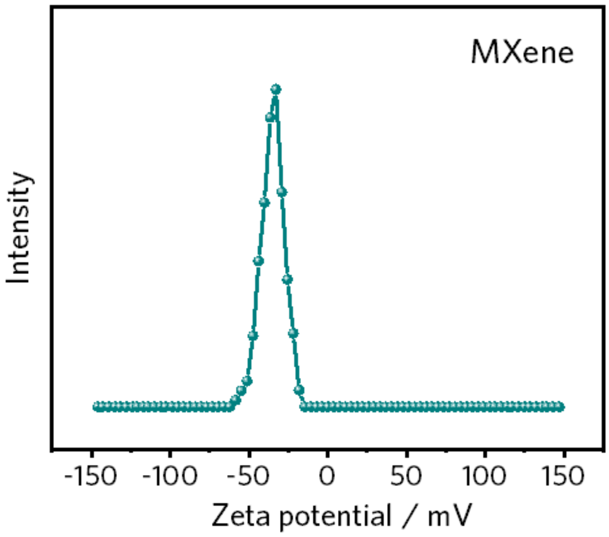

Fig. S2 Zeta potential of $Ti_3C_2T_x$ MXene nanosheets

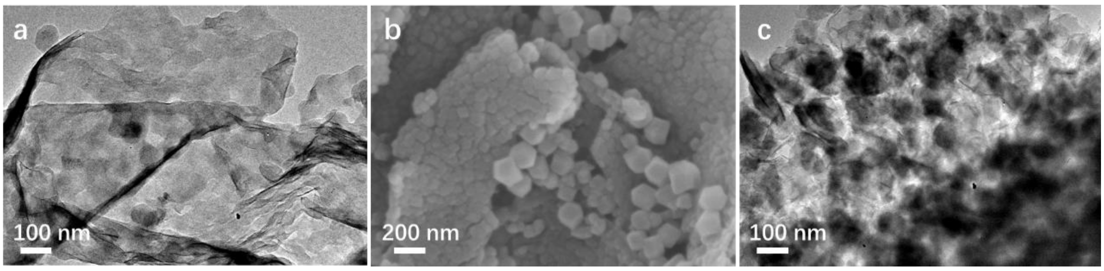

Fig. S3 TEM image of ZIF-8/MXene-0.9 (**a**), SEM image (**b**) and TEM image (**c**) of the ZIF-8/MXene-2

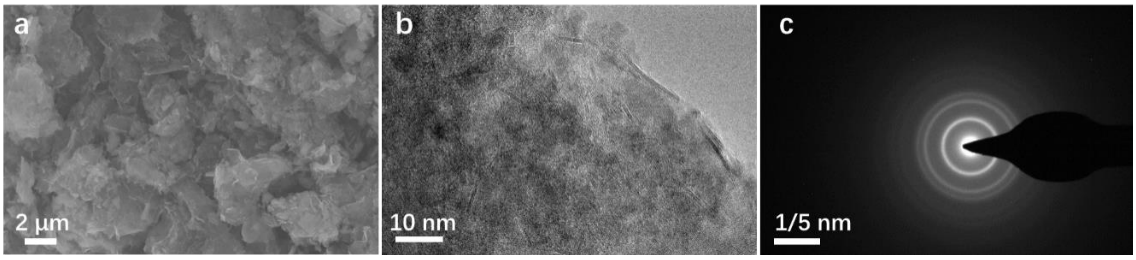

Fig. S4 SEM image (**a**) and TEM image (**b**) with the corresponding SAED pattern (**c**) of ZnSMX80

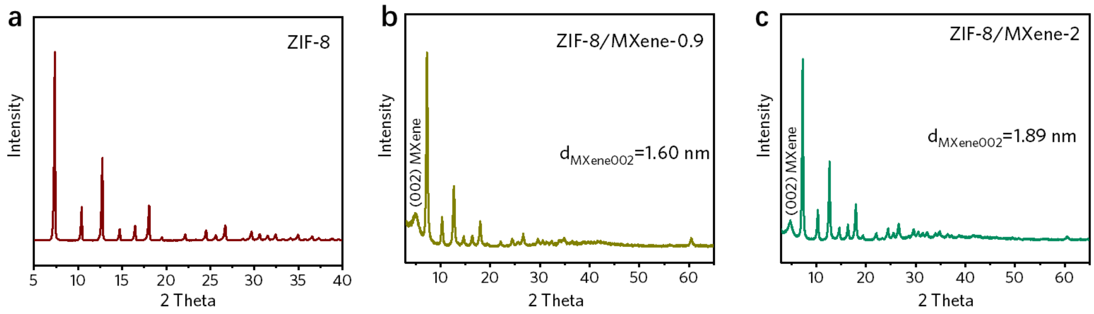

Fig. S5 XRD patterns of ZIF-8 (**a**), ZIF-8/MXene-0.9 (**b**) and ZIF-8/MXene-2 (**c**)

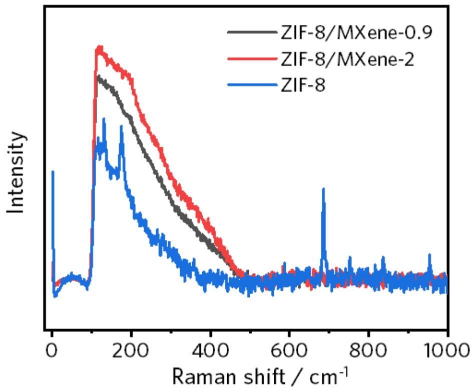

Fig. S6 Raman spectra of ZIF-8 and ZIF-8/MXene composites

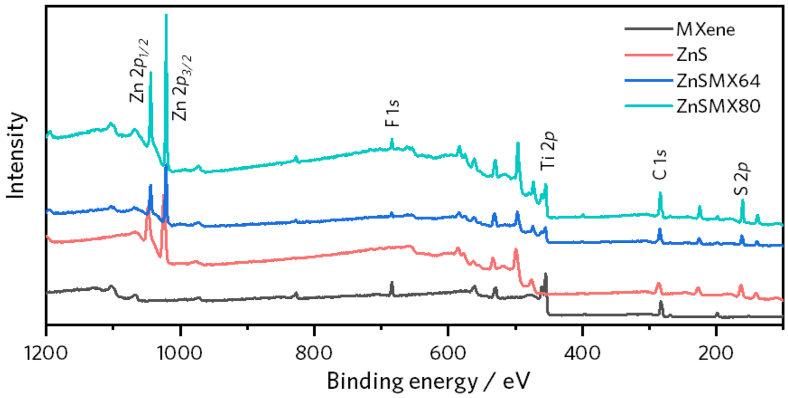

Fig. S7 XPS spectra of MXene, ZnS, ZnSMX64 and ZnSMX80

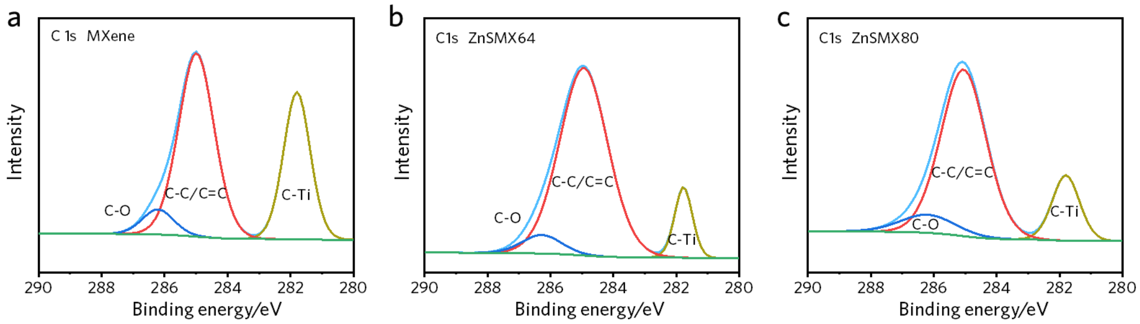

Fig. S8 High-resolution XPS spectra of C 1*s* for MXene (**a**), ZnSMX64 (**b**), and ZnSMX80 (**c**)

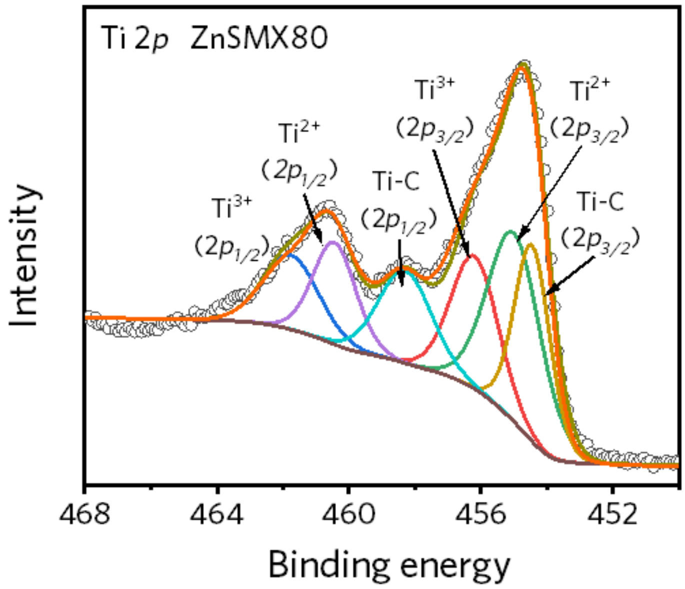

Fig. S9 High-resolution XPS spectra of Ti 2*p* for ZnSMX80

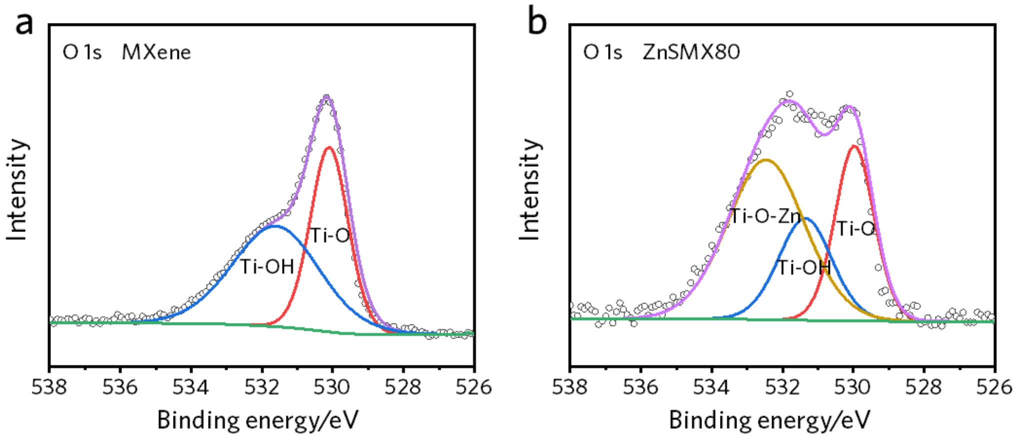

Fig. S10 High-resolution XPS spectra of O 1*s* for MXene (**a**) and ZnSMX80 (**b**)

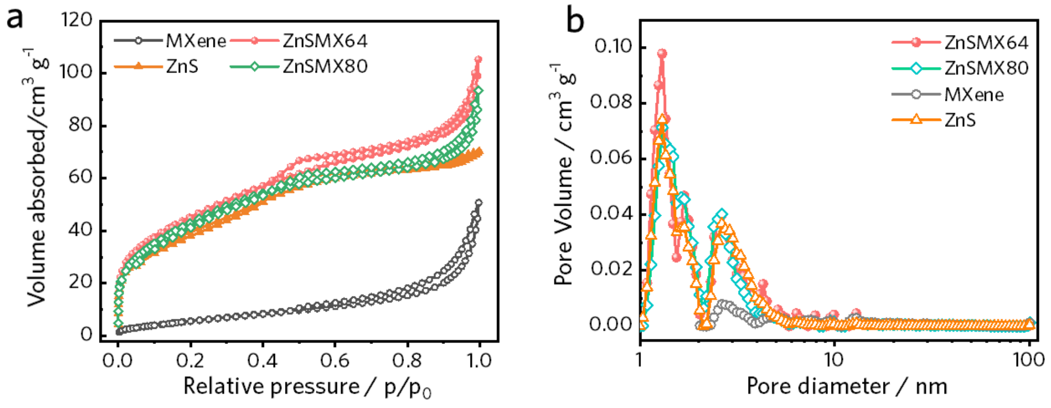

Fig. S11 Nitrogen (77K) adsorption-desorption isotherms (**a**) and pore size distributions (**b**) of all the samples

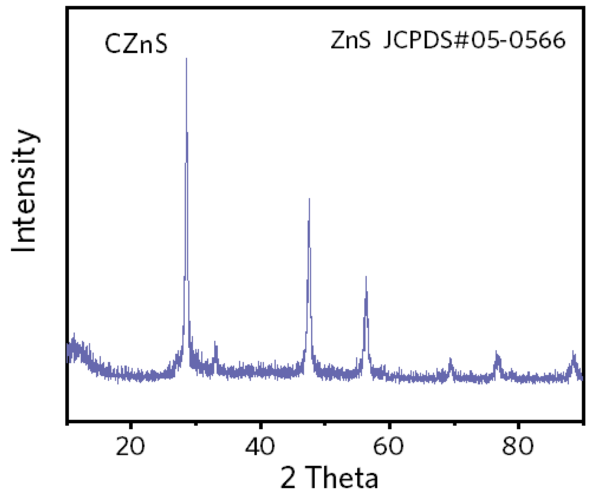

Fig. S12 XRD pattern of CZnS

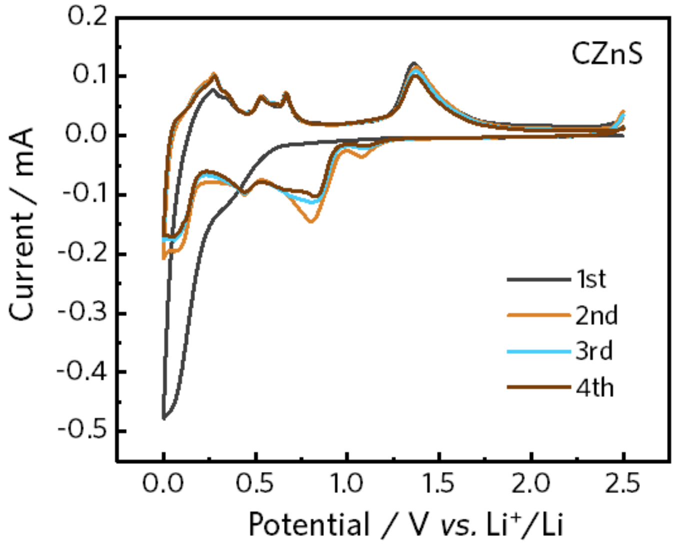

Fig. S13 CV curves of CZnS anode

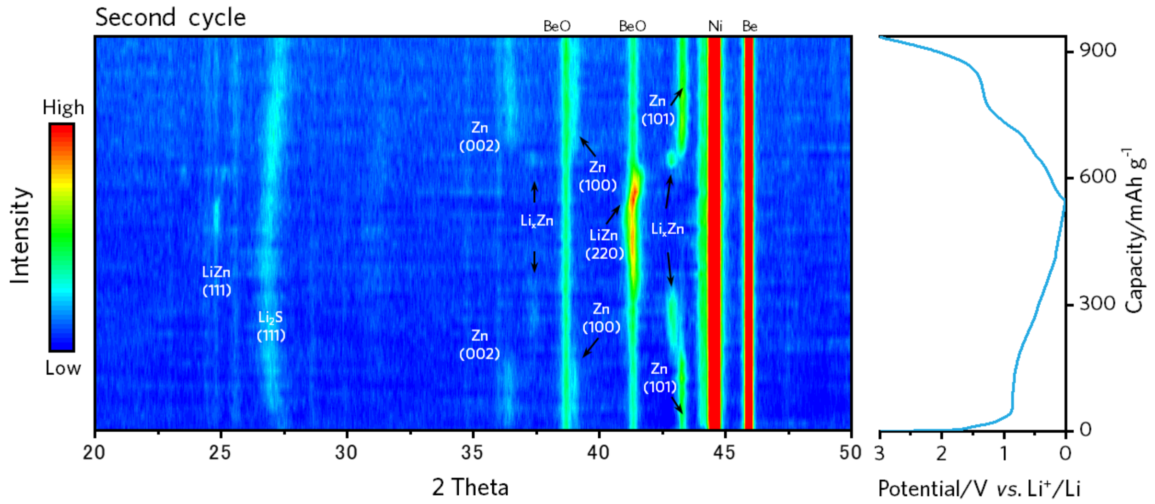

Fig. S14 Contour maps of *in situ* XRD characterization during second cycle for CZnS anode

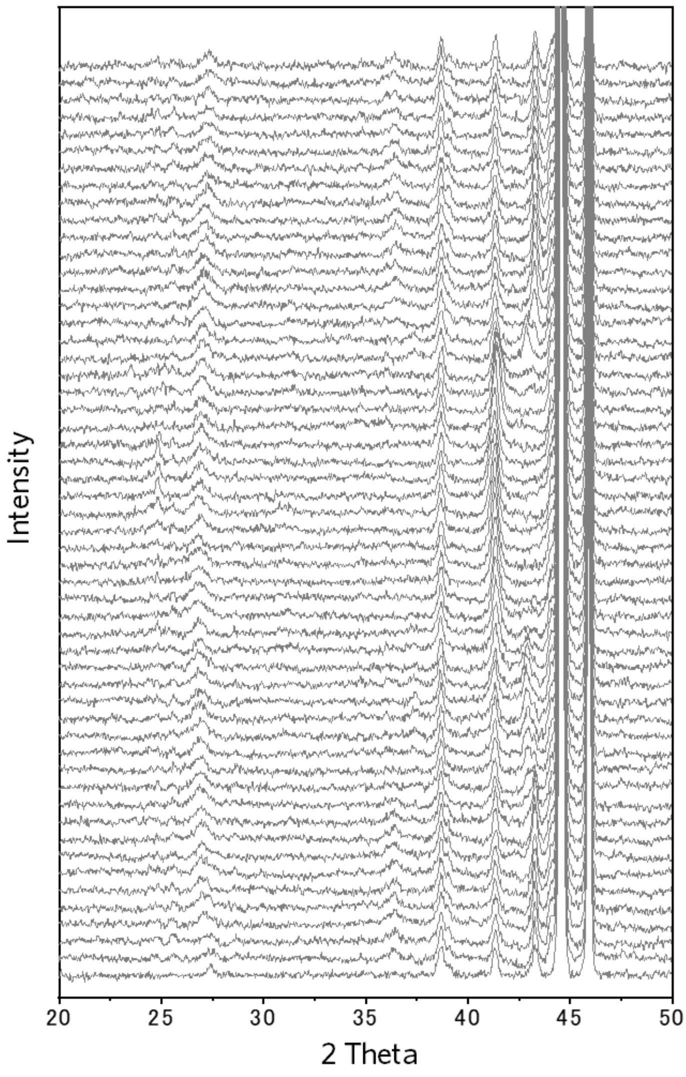

Fig. S15 *In situ* XRD patterns for the second cycle of CZnS anode

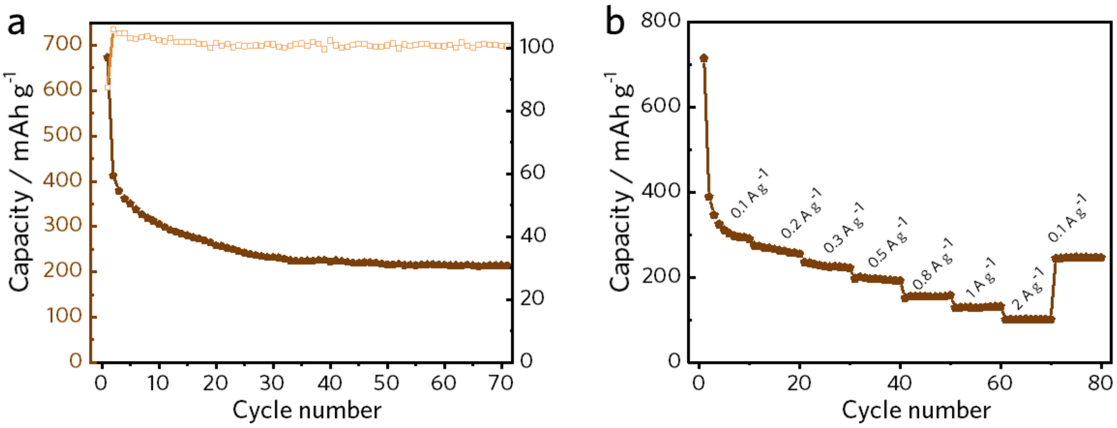

Fig. S16 Cycle performance (**a**) and rate capability (**b**) of CZnS anode

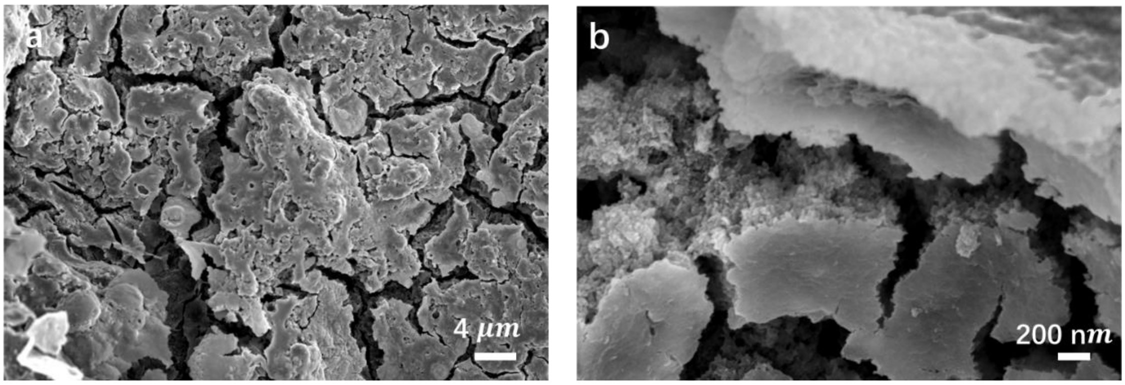

Fig. S17 SEM images of CZnS anode after 70 charge-discharge cycles

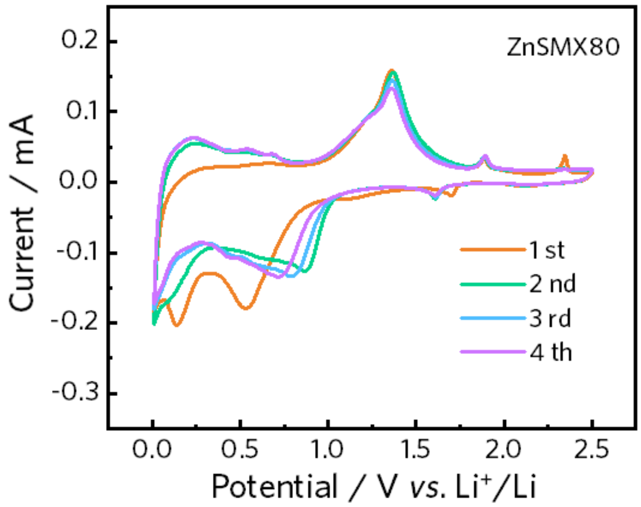

Fig. S18 CV curves of ZnSMX80 at 0.1 mV s^{-1}

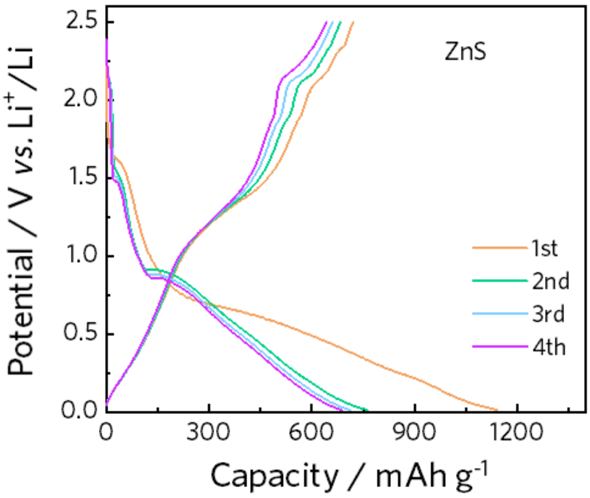

Fig. S19 Galvanostatic discharge-charge curves at 0.1 A g^{-1} for ZnS

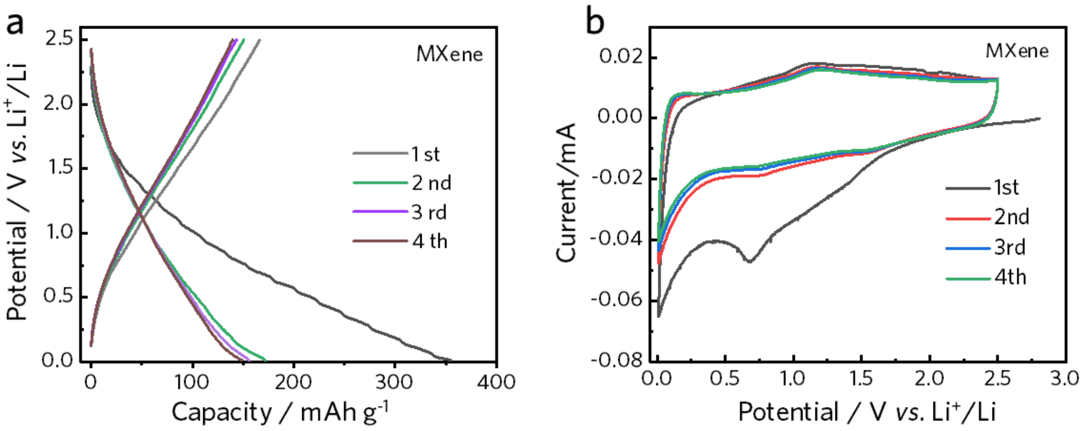

Fig. S20 Electrochemical performance of MXene. Galvanostatic discharge-charge curves at a current density of 0.1 A g^{-1} (**a**) and CV curves at a scan rate of 0.1 mV s^{-1} (**b**)

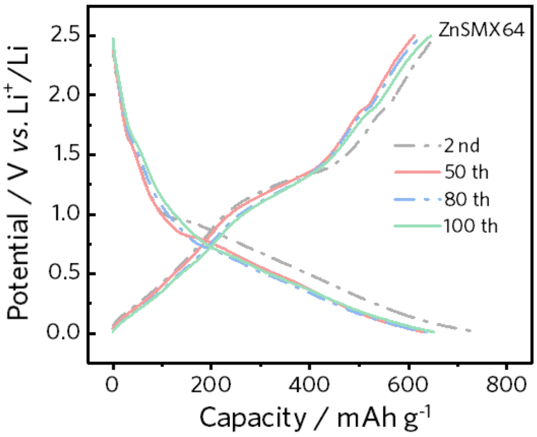

Fig. S21 Representative discharge-charge profiles of ZnSMX64 at 100 mA g^{-1} for 100 cycles

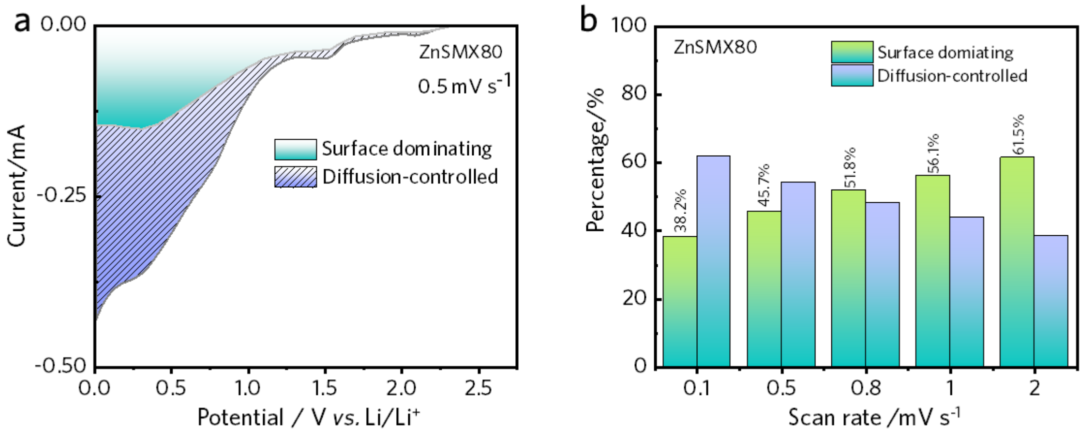

Fig. S22 CV curve of ZnSMX80 with the surface dominating capacity contribution for cathodic process at 0.5 mV s^{-1} (**a**), and the proportion of capacity contributions at different scan rates (**b**)

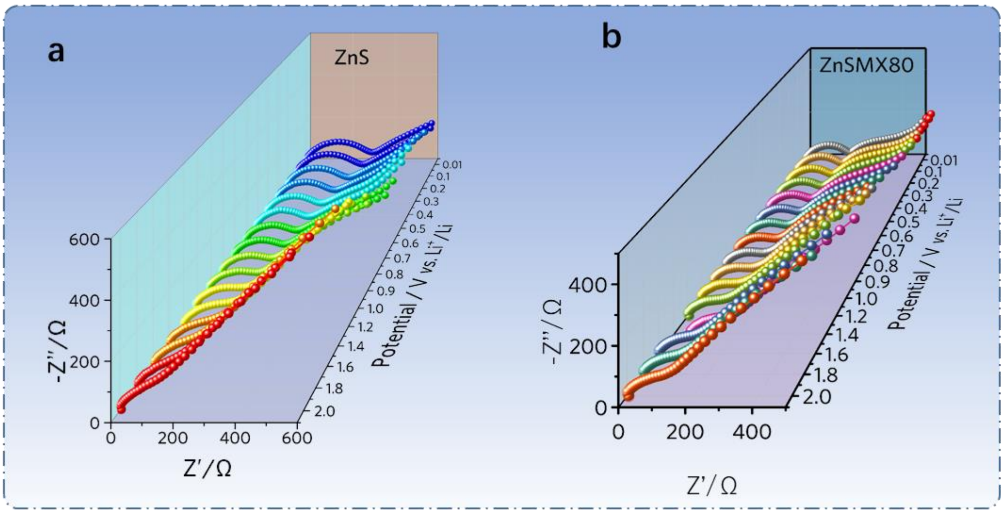

Fig. S23 *In situ* EIS characterization of ZnS (**a**) and ZnSMX80 (**b**) anode at different lithiation states of 2.0-0.01 V

The diffusion coefficient (D) can be calculated from the GITT potential profiles by Fick's second law with Eq. S1:

$$D = \frac{4}{\pi\tau}\left(\frac{m_B V_M}{M_B S}\right)^2\left(\frac{\Delta E_s}{\Delta E_\tau}\right)^2 \qquad \text{(S1)}$$

where τ is the titration time, m_B is the mass of active material, S is the area of electrodes, ΔE_s is the quasi-thermodynamic equilibrium potential difference before and after the current pulse, ΔE_τ is the potential difference during current pulse, V_M is the molar volume, and M_B is the molar mass.

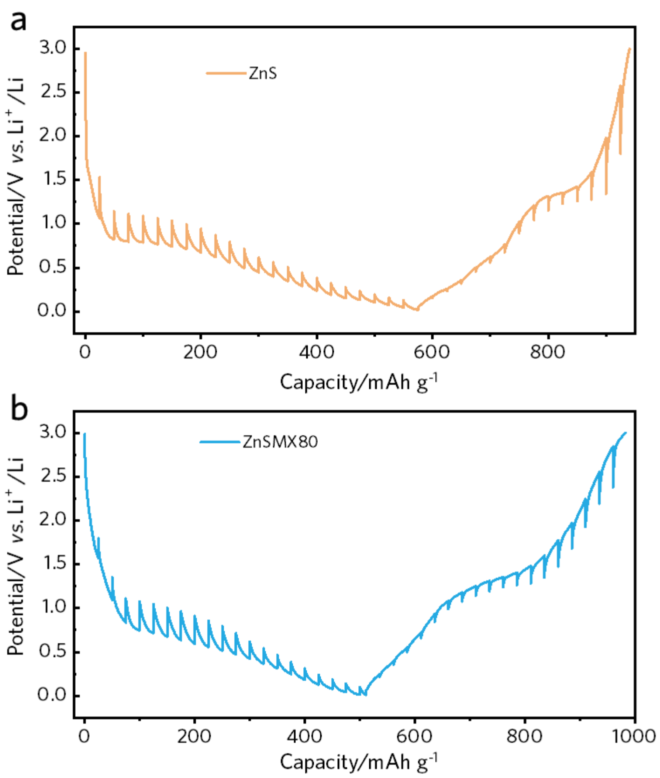

Fig. S24 GITT potential profile of the ZnS (**a**) and ZnSMX80 (**b**)

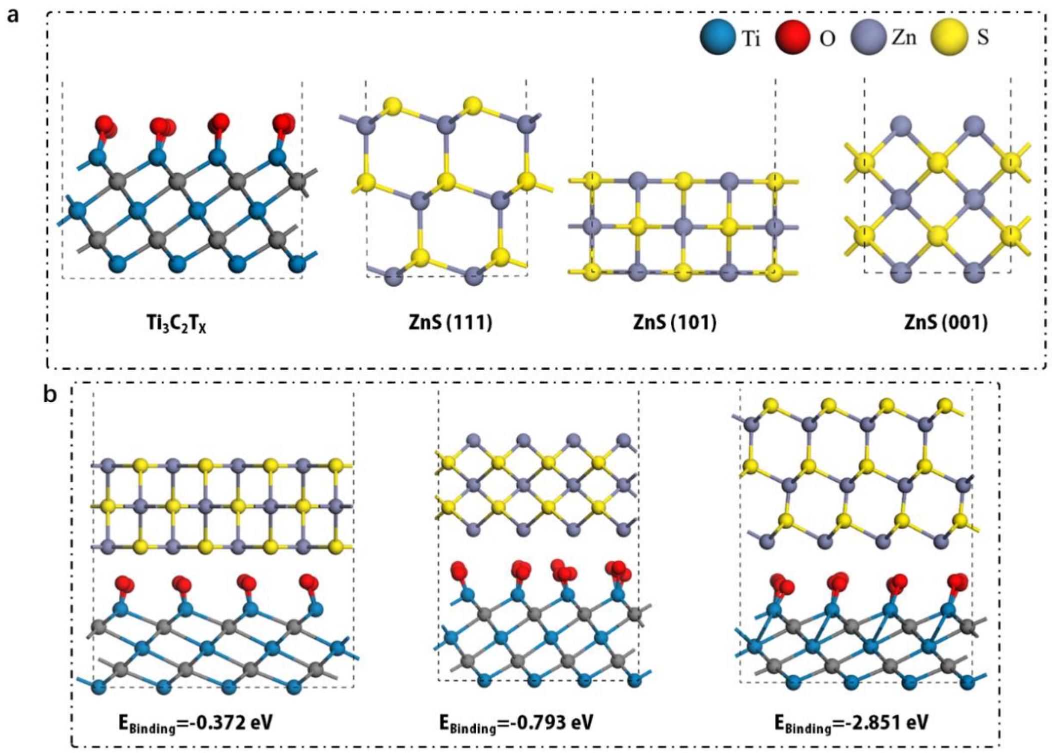

Fig. S25 (**a**) Simulation model of $Ti_3C_2T_x$, ZnS (111), ZnS (101) and ZnS (001). (**b**) Binding energy for ZnS (101)/$Ti_3C_2T_x$, ZnS (001)/$Ti_3C_2T_x$, and ZnS (111)/$Ti_3C_2T_x$. According to binding energy values of different structure, the ZnS (111)/$Ti_3C_2T_x$ structure is most stable

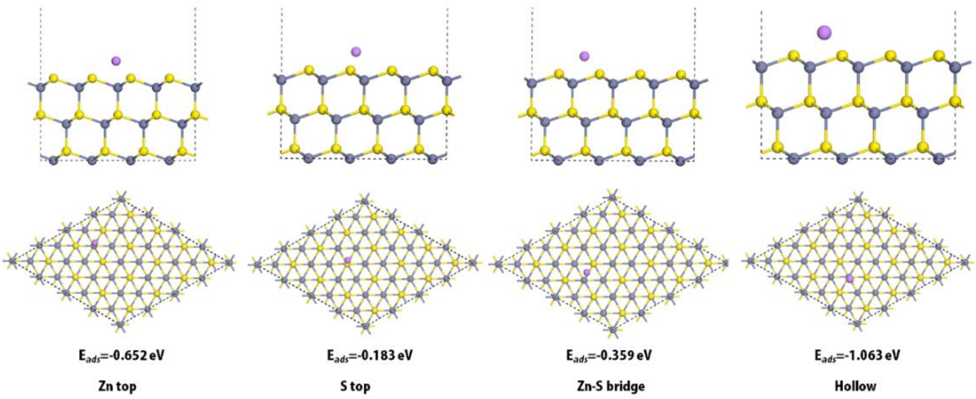

Fig. S26 Lithium adsorption in ZnS (111) model at Zn top, S top, Zn-S bridge, and hollow site with the corresponding adsorption energy

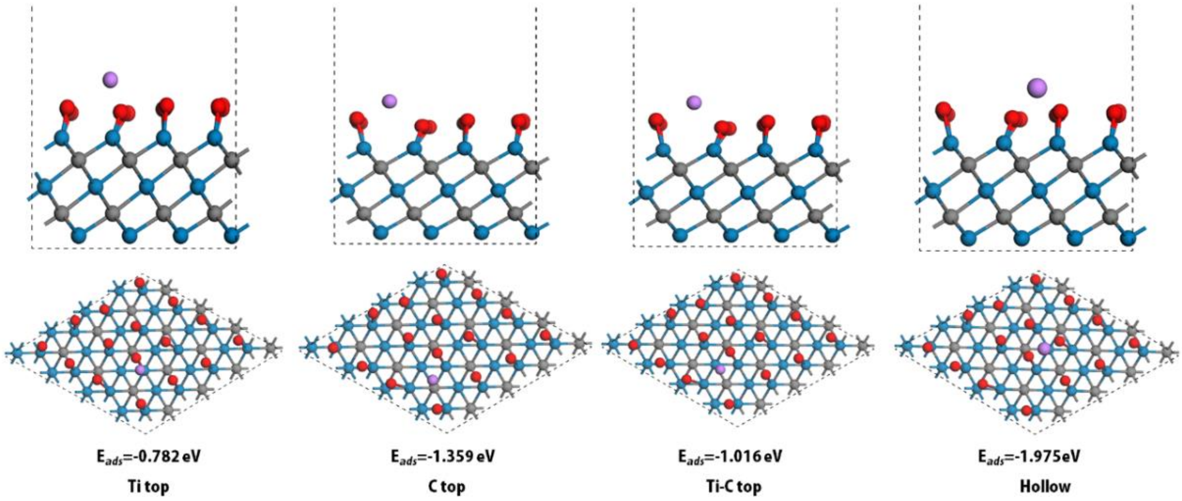

Fig. S27 Lithium adsorption in $Ti_3C_2T_x$ MXene model at Ti top, C top, Ti-C top and hollow site with the corresponding adsorption energy

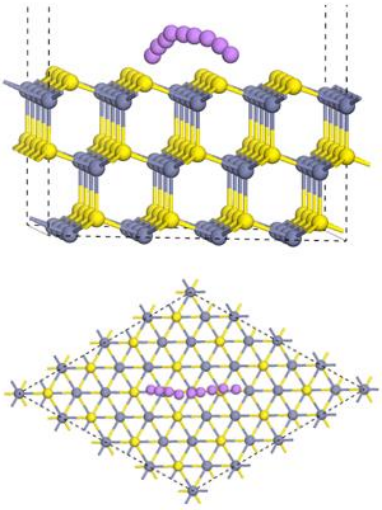

Fig. S28 Lithium diffusion path at ZnS (111)

Supplementary References

[S1] G. Kresse, J. Furthmuller, Efficiency of ab-initio total energy calculations for metals and semiconductors using a plane-wave basis set. Comput. Mater. Sci. **6**, 15 (1996). https://doi.org/10.1016/0927-0256(96)00008-0

[S2] G. Kresse, J. Furthmuller, Efficient iterative schemes for ab initio total-energy calculations using a plane-wave basis set. Phys. Rev. B **54**(16) 11169 (1996). https://doi.org/ 10.1103/PhysRevB.54.11169

[S3] K.B. John P. Perdew, Ernzerhof Matthias, Generalized gradient approximation Made Simple. Phys. Rev. Lett. **77**, 3865 (1996). https://doi.org/10.1103/PhysRevLett.77.3865

[S4] G.K.D. Joubert, From ultrasoft pseudopotentials to the projector augmented-wave method. Phys. Rev. B **59**, 1758 (1998). https://doi.org/10.1103/PhysRevB.59.1758
